# Principal Component Analysis of HPLC Retention Data and Molecular Modeling Structural Parameters of Cardiovascular System Drugs in View of Their Pharmacological Activity

**DOI:** 10.3390/ijms11072681

**Published:** 2010-07-09

**Authors:** Jolanta Stasiak, Marcin Koba, Leszek Bober, Tomasz Bączek

**Affiliations:** 1 Department of Medicinal Chemistry, Faculty of Pharmacy, Collegium Medicum, Nicolaus Copernicus University, Bydgoszcz, Poland; E-Mails: jola.stasiak@cm.umk.pl (J.S.); kobamar@cm.umk.pl (M.K.); 2 Polpharma SA Pharmaceutical Works, Starogard Gdański, Poland; E-Mail: leszek.bober@plusnet.pl; 3 Department of Pharmaceutical Chemistry, Faculty of Pharmacy, Medical University of Gdańsk, Gdańsk, Poland

**Keywords:** high-performance liquid chromatography (HPLC), cardiovascular system drugs, principal component analysis (PCA), molecular modeling parameters

## Abstract

Evaluation of relationships between molecular modeling structural parameters and high-performance liquid chromatography (HPLC) retention data of 11 cardiovascular system drugs by principal component analysis (PCA) in relation to their pharmacological activity was performed. The six retention data parameters were determined on three different HPLC columns (Nucleosil C18 AB with octadecylsilica stationary phase, IAM PC C10/C3 with chemically bounded phosphatidylcholine, and Nucleosil 100-5 OH with chemically bounded propanodiole), and using isocratically acetonitrile: Britton-Robinson buffer as the mobile phase. Additionally, molecular modeling studies were performed with the use of HyperChem software and MM+ molecular mechanics with the semi-empirical AM1 method deriving 20 structural descriptors. Factor analysis obtained with the use of various sets of parameters: structural parameters, HPLC retention data, and all 26 considered parameters, led to the extraction of two main factors. The first principal component (factor 1) accounted for 44–57% of the variance in the data. The second principal component (factor 2) explained 29–33% of data variance. Moreover, the total data variance explained by the first two factors was at the level of 73–90%. More importantly, the PCA analysis of the HPLC retention data and structural parameters allows the segregation of circulatory system drugs according to their pharmacological (cardiovascular) properties as shown by the distribution of the individual drugs on the plane determined by the two principal components (factors 1 and 2).

## 1. Introduction

The cardiovascular system, which distributes blood and provides the nutrients the system needs to keep the heart pumping, is a closed system (meaning that the blood never leaves the network of arteries, veins and capillaries), The main components of this system are the heart, the blood, and the blood vessels [1].

Cardiovascular (cardiac) pharmaceutical agents are divided in groups of drugs such as antiarrhythmic agents, ACE inhibitors, angiotensin II receptor antagonists, beta blocker, calcium channel blocker, and drugs with blood vassels activity [1–3]. The antiarrhythmic group of pharmaceuticals agents are used to suppress fast rhythms of the heart (cardiac arrhythmias), such as atrial fibrillation, atrial flutter, ventricular tachycardia, and ventricular fibrillation. There are five main classes of antiarrhythmic agents proposed by Vaughan Williams (VW): class I agents interfere with the sodium (Na^+^) channel, divided additionally into subclass Ia-c (drugs such as dispyramide, quinidine, phenytoine, propaphenone, *etc*.), class II consists of anti-sympathetic nervous system agents with most agents in this class functioning as beta blockers (e.g., metopropolol, *etc*.), class III agents affect potassium (K^+^) efflux (e.g., amidarone, sotalol, *etc*.), class IV agents affect calcium (Ca^2+^) channels (e.g., diltiazem, verapamil, *etc*.), and class V agents work by other or unknown mechanisms (drugs such as adenosine, digoxin, *etc*.). This classification is based on the primary mechanism of antiarrhythmic effect. However, some of antiarrhythmic agents (for example, amiodarone) have multiple mechanisms of action [1,2,4–6]. The group of ACE inhibitors (angiotensin-converting enzyme inhibitors) is a group of pharmaceuticals (compounds such as captopril, perindopril, *etc*.) that are used primarily in the treatment of hypertension and congestive heart failure, and in some cases as the drugs of first choice. Generally, ACE inhibitors block the conversion of angiotensin I to angiotensin II, and consequently lower arteriolar resistance and increase venous capacity, increase cardiac output and cardiac index, lower renovascular resistance, and increase natriuresis [1,7]. On the other hand, angiotensin II receptor antagonists, also known as angiotensin receptor blockers (ARBs), are a group of pharmaceuticals (drugs such as candesartan, irbesartan, *etc*.) that modulate the renin-angiotensin-aldosterone system. They block the activation of angiotensin II receptors via block of AT_1_ receptors, causing vasodilation, reduced of secretion of vasopressin, reduced production and secretion of aldosterone, and reduction of blood pressure. Their main use is in hypertension (high blood pressure), diabetic nephropathy (kidney damage due to diabetes) and congestive heart failure. Moreover, angiotensin II receptor antagonists are primarily used for the treatment of hypertension when the patient is intolerant to ACE inhibitor therapy [1,8]. The beta blockers (β-blockers) is a class of drugs (compounds such as acebutolol, atenolol, carvedilol, nadolol, butaxamine, *etc*.) used for various indications, but particularly for the management of cardiac arrhythmias, cardioprotection after myocardial infarction (heart attack), and hypertension. There are three known types of beta receptor, designated β_1_, β_2_ and β_3_ [1,9]. The calcium channel blockers (CCBs) are a class of drugs and natural substances (compounds such as amlodipine, verapamil, diltiazem, *etc*.) that disrupt the calcium (Ca^2+^) conduction of calcium channels, and have effects on many cells such as cardiac muscle, *i.e.*, heart, smooth muscles of blood vessels, or neurons. They main clinical usage is to decrease blood pressure. Additionally, the calcium channel blockers are further divided into some classes such as dihydropyridine, phenylalkylamine or benzothiazepine calcium channel blockers. The dihydropyridine calcium channel blockers (drugs as amlodipine, nifedipine, nimodipine, nisoldipine, nitrendipine and others) are often used to reduce systemic vascular resistance and arterial pressure, but are not used to treat angina because the vasodilation and hypotension can lead to reflex tachycardia, compared to phenylalkylamine calcium channel blockers (drugs such as verapamil and others) which are relatively selective for myocardium, reduce myocardial oxygen demand and reverse coronary vasospasm, and are often used to treat angina. They also have minimal vasodilatory effects compared with dihydropyridines and therefore cause less reflex tachycardia, making it appealing for treatment of angina. However, the class of benzothiazepine calcium channel blockers (drugs such as diltiazem, *etc*.) is an intermediate class between phenylalkylamine and dihydropyridines in their selectivity for vascular calcium channels. Moreover, by having both cardiac depressant and vasodilator actions, benzothiazepines are able to reduce arterial pressure without producing the same degree of reflex cardiac stimulation caused by dihydropyridines [1,9–12]. On the other hand, the drugs with blood vassels activity (compounds such as pentoxphylline, *etc*.) improve blood flow through blood vessels and therefore help with blood circulation in the arms and legs, and also help to prevent stroke, can be used in managing sickle cell disease and improve blood flow to the brain. They are also used to treat intermittent claudication resulting from obstructed arteries in the limbs, and vascular dementia [1,13–15].

Principal component analysis (PCA) is a method intending to extract and visualize systematic patterns or trends in large data matrices. By PCA, one can reduce the number of variables in a data set by finding linear combinations of variables explaining most of the variability. It is known that the independent variables in multiple regression analysis are often mutually inter-correlated and therefore are inappropriate for that kind of analysis. On the other hand, such variables can be subjected to multivariate analysis, as for example PCA or factor analysis. By PCA, all those original parameters which are interrelated by simple or multiple correlations can be combined in a linear manner to the limited number of orthogonal principal components (factors) [16]. The PCA method as so far was used for the pharmacological classification of a large set of drugs based on HPLC retention data [17–21]. Recently, PCA of HPLC retention data in combination with molecular modeling structural parameters found a wide application in QSAR analysis for pharmacological classification of drugs [22].

The goal of the present study was to determine the relationships between HPLC retention parameters of a series of compounds differing in chemical structure and characterized by cardiac pharmacological activity and their structural parameters obtained by molecular modeling calculations applying the PCA method. The following 11 cardiovascular system drugs were selected for the proposed studies: amiodarone, amlodipine, nifedipine, nimodipine, nisoldipine, nitrendipine, verapamil, diltiazem, disopyramide, propaphenone and pentoxyphylline.

## 2. Results and Discussion

**Figure 1.**
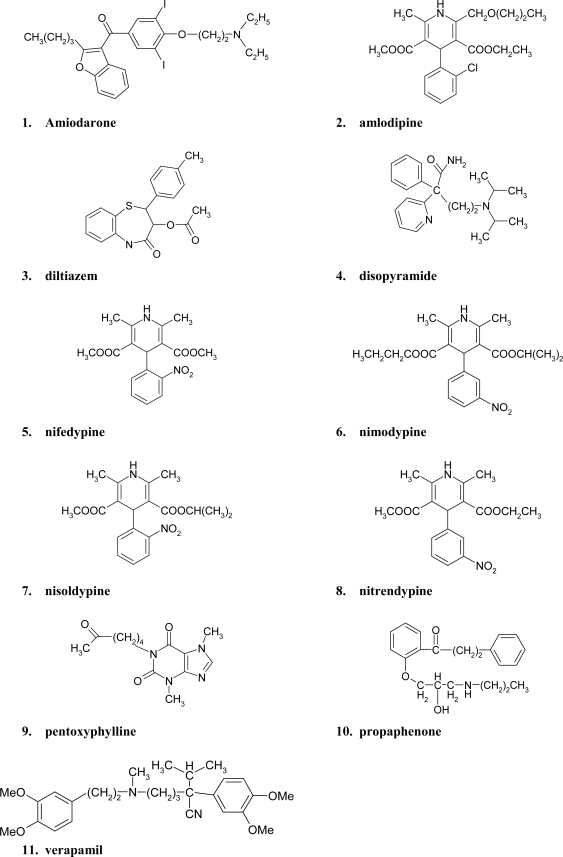
Chemical structures of the 11 studied compounds.

**Figure 2.**
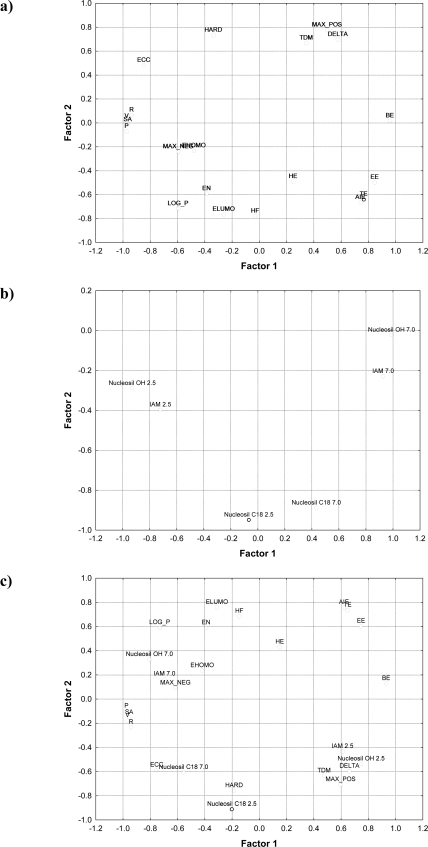
Two-dimensional scatter plots of the loadings of the first two factors: **a)** by structural parameters, **b)** by HPLC retention data, **c)** by structural parameters along with HPLC retention data.

The chemical structures of the studied compounds and their pharmacological classification are presented in Figure 1 and Table 1, respectively. The values of six HPLC retention parameters (Nucleosil C18 2.5- Nucleosil OH 7.0) and 20 structural parameters (TE-P) for the considered compounds are presented in Table 2. Factor analysis led to the extraction of four, three or five main factors with eigenvalue higher than 1 from the analyzed group of parameters: structural parameters, HPLC retention data and all parameters, respectively (for results see Table 3). In the set of structural parameters, the first factor accounted for 46% of the data variance and the second one for 29%. On the other hand, in the set of HPLC retention data and all 26 analyzed parameters, the first factor accounted for 57% and 44% of the data variance, respectively, and second one for 33% and 29%, respectively. These data indicate that the majority of the information contained in the original data matrix can be explained by two principal components and it can be interpreted that two principal components contain the significance part of information held previously in original molecular descriptors (about 75% of information), HPLC retention (about 90% of information) or altogether HPLC and molecular properties variables (about 73% of information). Additionally, the results of factor analysis that represent the first two loadings (factors 1 and 2) of each variables and their two-dimensional scatter plots obtained with the use of various sets of parameters (structural parameters, HPLC retention data, and all 26 parameters from structural parameters and HPLC retention data) were collected in Table 4 and Figure 2a, Table 5 and Figure 2b, and Table 6 and Figure 2c, respectively. The highest factor loadings among the variables over 0.7 were presented in bold type. Moreover, in the set of structural parameters (Figure 2a) the factor 1 depended mostly on total energy (TE), binding energy (BE), atom interaction energy (IAE), electronic energy (EE), core-core interaction energy (ECC), surface area of the molecule available for solvent (SA), volume of molecule (V), refraction (R), polarizability (P), whereas factor 2 depended mostly on heat of formation (HF), lowest unoccupied molecular orbital energy (ELUMO), the “hardness” of molecules (HARD), the value of the highest positive charge of atoms that constitute a molecule (MAX_POS), the difference between the highest positive and negative charges of atoms constituting a molecule (DELTA), and the logarithm of the *n*-octanol-water partition coefficient (LOG_P). The presented data are in accordance with our previous results [22] obtained for some antipyretic, anti-inflammatory and analgesic drugs, and showed that factor 1 presented mainly properties connected with molecular (size) bulkiness (like SA, V, R, P or TE), whereas factor 2 presented properties related to electronic propertied (like ELUMO, MAX_POS, or DELTA).

In the case of the HPLC retention dataset (Figure 2b), factor 1 depended on log k parameters obtained on columns packed with stationary phases other than octadecylsilica such as IAM PC C10/C3 column with chemically bounded phosphatidylcholine and Nucleosil 100-5 OH with chemically bounded propanodiole at both 2.5 and 7.0 pH. On the other hand, factor 2 depended on log k_w_ parameters obtained on a Nucleosil C18 AB column packed with octadecylsilica also at both 2.5 and 7.0 pH. However, these results are contrary to data obtained for some antipyretic, anti-inflammatory and analgesic drugs [22], which showed that factor 1 depended mostly on chromatographically data (log k_w_) obtained only on a Nucleosil C18 AB column, whereas factor 2 depended mainly on log k_w_ parameters obtained on columns packed with stationary phases other than octadecylsilica.

In the dataset including all parameters (Figure 2c), factor 1 depended mostly on log k values obtained on IAM PC C10/C3 column only at pH 7.0 and on Nucleosil 100-5 OH column at both pH 2.5 and 7.0 in the set of HPLC retention parameters, and on binding energy (BE), electron energy (EE), core-core interaction energy (ECC), surface area of the molecule available for solvent (SA), volume of molecule (V), logarithm of the *n*-octanol-water partition coefficient (LOG_P), refraction (R) and polarizability (P) in the set of molecular parameters. It is evident that these parameters reflect the size (bulkiness) of compounds studied, and condenses mainly information about their molecular size. On the other hand, the factor 2 depended mostly on log k_w_ value obtained on Nucleosil C18 AB column only at pH 2.5 in the set of HPLC retention parameters, and on total energy (TE), atom interaction energy (IAE), lowest unoccupied molecular orbital energy (ELUMO), the “hardness” of molecules (HARD) and the value of the highest positive charge of atoms that constitute a molecule (MAX_POS) in the set of molecular parameters. In this case, factor 2 presented properties related to electronic properties rather than their bulkiness.

**Figure 3.**
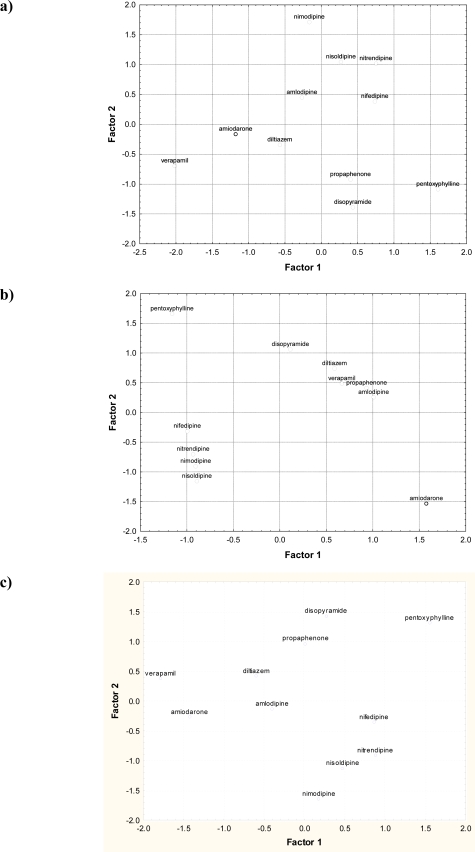
Two-dimensional scatter plots of the scores of individual drugs in the first two factors extracted: **a)** from structural parameters, **b)** from HPLC retention data, **c)** from structural parameters and HPLC retention data.

Almost all the information (total data variance at the level 73–90%) can be explained by the first two principal components. Therefore, specific compounds can be compared on the basis of two principal component scores (objects) plots. Principal component scores calculated for all studied compounds and their individual positions on the plane determined by the two factor axes and performed only for structural parameters, only for HPLC retention data, and for all structural and HPLC retention data parameters together are presented in Table 7, and Figure 3a–c, respectively. Moreover, most of the studied compounds possess various pharmacological properties (antiarrhythmic or non- antiarrhythmic activity, blood vassels activity, or antihypertension activity), and their activities in all these aspects should be estimated in the same conditions. However, the classification of studied cardiovascular system drugs according to their pharmacological properties based on literature data is presented in Table 1.

Figure 3a presents the positions of particular compounds on the plane determined by factors 1 and 2 obtained for structural parameters, and is characterized by an arrangement of three clusters. The first cluster contains disopyramide and propaphenone with about a value of 0.5 of factor 1 and with negative values of factor 2. These compounds are sodium class I channel blockers and are characterized by antiarrhythmic activity (Table 1). The second and scattered cluster form amlodipine, nifedipine, nimodipine, nisoldipine, nitrendipine, with positive values of factor 2 as dihydropyridine derivative calcium channel blockers without antiarrhythmic activity (Table 1)and belong chemically to 4-phenyl-6-methyl-1,4-dihydropyridine-3,5-dicarboxylate derivatives. Another cluster on the scatter diagram in Figure 3a comprises verapamil, amiodarone and diltiazem, with negative values of factor 1 and factor 2, and characterized by antiarrhythmic channel blockers activity (Table 1). However, on the scatter diagram, the drug pentoxyphylline (with negative values of factor 1 and the most positive values of factor 2) is characterized only by blood vassels activity, and not classified to any of the three pharmacologically-related clusters proposed above.

The positions of particular compounds on the plane determined by factors 1 and 2 obtained by HPLC retention data is presented in Figure 3b. On the scatter diagram, two main and close clusters were observed. The first cluster, with negative values of factor 1 factor 2, contains nifedipine, nimodipine, nisoldipine, nitrendipine as 2,6-dimethyl-4-phenyl-1,4-dihydropyridine-3,5-dicarboxylate derivatives with a nitro- group attached to position 2 or 3 of phenyl moiety, and belongs pharmacologically to the dihydropyridine class selective calcium channel blockers with lack of antiarrhythmic activity and additionally characterized by antihypertension activity (Table 1). The second cluster, with positive values of factor 1 factor 2, formed by diltiazem, verapamil, propaphenone, amlodipine and disopyramide further away, and linked compounds with sodium or calcium channel blockers activity and antiarrhythmic activity (exclude amlodipine with lack of antiarrhythmic activity), and also antihypertension activity (Table 1). However, there are two drugs, pentoxyphylline and amiodarone, not classified to the two proposed main clusters. The latter compound belongs to α-receptors and potassium channel blockers with antiarrhythmic activity, whereas pentoxyphylline is characterized only by blood vassels activity.

The position of particular compounds on the plane determined by factors 1 and 2 obtained for all 26 parameters is presented in Figure 3c. On this scatter diagram, two main clusters are observed, however with some sub-clusters showing better differentiation (similarities and dissimilarities) in pharmacological features compared to the clusters presented in Figure 3a and b. The first main cluster, with positive values of factor 1 and negative values of factor 2, is formed by nifedipine, nimodipine, nisoldipine, nitrendipine, and belongs to the dihydropyridine class selective calcium channel blockers and is characterized by lack of antiarrhythmic activity (Table 1). The second main cluster, with near 0 and negative values of factor 1, and near 0 and positive values of factor 2, is formed by diltiazem, verapamil, propaphenone, amlodipine, disopyramide and amiodarone, and is characterized by antiarrhythmic activity (exclude amlodipine with lack of antiarrhythmic activity) (Table 1). Additionally, some compounds from this main cluster form other small sub-clusters containing, first, disopyramide and propaphenone as drugs belongs to class I sodium channel blockers, and second, containing diltiazem and amlodipine as calcium channel blockers (Table 1). The other small cluster contains compounds verapamil and amiodarone, belonging to the class of potassium or calcium channel blockers as well as the class of α-receptors.

## 3. Materials and Methods

### 3.1. Drugs

In all experiments, the following drugs were investigated: amiodarone (1), diltiazem (3), disopyramide (4), nifedypine (5), pentoxyphylline (9), propaphenazone (10) and verapamil (11) all from Polpharma S.A., Starogard Gdański, Poland; amlodypine (2) from Pfizer US Pharmaceutical Group, New York, NY, USA; nimodypine (6), nisoldypine (7) and nitrendypine (8) all from Bayer, Leverkusen, Germany.

### 3.2. Chromatographic Conditions

Chromatographic analysis was performed with a Waters SM 2690 Alliance HPLC system equipped with a PDA 996 diode detector (Waters Corporation, Milford, MA, USA) and Compaq Deskpro computer (Compaq Computer Corporation, Houston, TX, USA) with the Millennium 3.2 program for data collection and the process control. The following HPLC columns were employed: (a) Nucleosil C18 AB column, 50 × 3.0 mm i.d. (Macherey-Nagel, Düren, Germany), packed with octadecylsilica with particles size 5 μm; (b) IAM PC C10/C3 column, 150 × 4.6 mm i.d. (Regis Chemical Company, Morton Grove, IL, USA), packed with silica propylamine with the unreacted propylamine moieties endcapped with methylglycolate, and chemically bounded phosphatidylcholine, with particles size 12 μm; (c) and Nucleosil 100-5 OH column, 250 × 4.0 mm i.d. (Macherey-Nagel, Düren, Germany), packed with silica gel with chemically bounded propanodiole, with particles size 5 μm.

The compounds studied were chromatographed applying isocratic conditions on the above mentioned columns at ambient temperature. The mobile phase was acetonitrile: Britton-Robinson buffer prepared at pH 2.5 and 7.0 by adding 0.2 M sodium hydroxide to a solution of 0.04 M acetic acid, 0.04 M phosphoric acid and 0.04 M boric acid. In the case of Nucleosil C18 AB column, the eluent was used with the following proportions 90:10, 80:20, 70:30, 60:40, 50:50, 40:60 and 30:70 (% *v/v*). However, in the case of IAM PC C10/C3 and Nucleosil 100-5 OH columns, experiments were performed for acetonitrile: Britton-Robinson buffer at pH 2.5 and 7.0 with the proportions 50:50 and 40:60 (% *v/v*), respectively. The detection wavelength was 254 nm. Additionally, all the mobile phases used in HPLC were filtered through a GF/F glass microfiber filter (Whatman, Maidstone, UK) and degassed by ultrasonication immediately before use. The compounds studied were dissolved in methanol.

The logarithm of the HPLC retention factors (log k) for particular chromatographed compounds were calculated in the case of IAM PC C10/C3 and Nucleosil 100-5 OH columns, and subjected further to factor analysis. In the case of Nucleosil C18 AB column, the logarithms of the HPLC retention factors (log k) for particular chromatographed compounds in the given chromatographic system were regressed against the volume fraction of organic modifier in the eluent. The linear part of relationship was extrapolated to a hypothetical retention factor corresponding to 0% of organic modifier in the mobile phase. The resulting retention parameters were normalized to pure buffer using linear extrapolation and defined as log k_w_ and subjected further to factor analysis.

### 3.3. Structural Parameters

The structures of the tested compounds were investigated by molecular modeling with the use of HyperChem 7.5 software (HyperCube Inc., Gainesville, FL, USA) [23]. First, the structures of the compounds were pre-optimized geometrically with the molecular mechanics force field procedure (with MM+ method). It allowed preparing structures for further optimization steps. The resulting structures were optimized then by means of the quantum-based method, namely semi-empirical AM1 method and applying the Polak-Ribiere algorithm with gradient limit of 0.01 kcal Å^−1^.

The following molecular descriptors were considered: total energy (TE), binding energy (BE), atom interaction energy (IAE), isolated atom energy (AIE), electronic energy (EE), core-core interaction energy (ECC), heat of formation (HF), highest occupied molecular orbital energy (EHOMO) and lowest unoccupied molecular orbital energy (ELUMO). Moreover, electronegativity (EN) was calculated as an arithmetic mean of ionization potential and electron affinity according to Mulliken [24,25]. The “hardness” of molecules (HARD) was calculated according to Parr and Pearson [26] as well as Robles and Bartolotti [27] and presented as half of the difference between the ionization potential and the electron affinity. Additionally, the following values were used: the values of the highest positive (MAX_POS) and negative (MAX_NEG) charge of atoms that constitute a molecule, the difference between the highest positive and negative charges of atoms constituting a molecule (DELTA) and total dipole moment (TDM).

Moreover, additional parameters were calculated using the QSAR Properties Module of HyperChem 7.5 software and include the following: surface area of the molecule available for solvent (SA), volume of molecule (V), hydratation energy (HE), the logarithms of the *n*-octanol-water partition coefficient (LOG_P), refraction (R) and polarizability (P).

### 3.4. Statistical Analysis

The chemometric analysis was performed with the use of Statistica 9.0 software (StatSoft, Tulsa, OK, USA) with the application of principal component analysis (PCA).

## 4. Conclusions

PCA was performed for structural parameters data and HPLC retention data obtained for 11 cardiovascular drugs. Based on the above discussion of results, the following conclusions may be proposed. The pattern of distribution of individual drugs on the plane determined by two principal components (factors 1 and 2) obtained on the basis of structural parameters, log k and log k_w_ values were in good agreement with their pharmacological features.

Moreover, PCA led to the extraction of four, three or five main factors with eigenvalue higher than 1 from the analyzed groups of structural parameters, HPLC retention data or all parameters, respectively. The highest statistical significance were for factor 1 and factor 2. The first principle component (factor 1) accounted for 44–57% of variance in the data, and second principal component (factor 2) explained 29–33% of data variance, indicating that the total data variance that could be explained by the first two factors was at the level of 73–90%.

From the 26 parameters used, those with the most influence on the factor values were all the chromatographic parameters and some structural parameters such as total energy (TE), binding energy (BE), atom interaction energy (IAE), isolated atom energy (AIE), electronic energy (EE), core-core interaction energy (ECC), heat of formation (HF), lowest unoccupied molecular orbital energy (ELUMO), the “hardness” of molecules (HARD), value of the highest positive (MAX_POS) charge of atoms that constitute a molecule, the difference between the highest positive and negative charges of atoms constituting a molecule (DELTA), surface area of the molecule available for solvent (SA), volume of molecule (V), the logarithms of the *n*-octanol-water partition coefficient (LOG_P), refraction (R) and polarizability (P).

The PCA analysis proposed for application of chromatographic data together with molecular modeling data may help in the preliminary revision of data structure (subclasses of similar objects and related variables) for the studied cardiovascular system drugs as well as drug candidates according to similarities in their pharmacological properties. It can be used as a data compression and visualization method, helping finally in the proper interpretation of the received data.

## Figures and Tables

**Table 1.** The classification of the 11 studied cardiovascular system drugs according to their pharmacological activity.

**Compound**	**Antiarrhythmic activity a**						**Lack of anti-arrhythmic activity b**	**Blood vassels activity c**	**Anti hypertension activity d**

	Receptor		Chanel blockers						

	M2 muscarinic antagonist	α-receptors	Na^+^ class I		K^+^ class III	Ca^2+^ class IV	Ca^2+^		
			Ia	Ic					
**amiodarone**	−	+	−	−	+	−	−	−	+
**amlodipine**	−	−	−	−	−	−	+	−	+
**diltiazem**	−	−	−	−	−	+	−	−	+
**disopyramide**	+	−	+	−	−	−	−	−	+
**nifedipine**	−	−	−	−	−	−	+	−	+
**nimodipine**	−	−	−	−	−	−	+	−	+
**nisoldipine**	−	−	−	−	−	−	+	−	+
**nitrendipine**	−	−	−	−	−	−	+	−	+
**pentoxyphylline**	−	−	−	−	−	−	−	+	−
**propaphenone**	−	−	−	+	−	−	−	−	+
**verapamil**	−	+	−	−	−	+	−	−	+

a data from [1–4];

b data from [1,2,9,11,28–34];

c data from [1,2,35–43];

d data from [1,2,44–46].

Antiarrhythmic activity, blood vassels activity and antihypertension activity are presented as positive (+) or negative (−).

**Table 2.** Values of HPLC retention data and molecular descriptors used in principal component analysis (PCA). See Materials and methods for HPLC column characteristics and definitions of molecular parameters.

**Compound**	**HPLC retention data**												**Molecular descriptors**													

	**Nucleosil C18 2.5**	**Nucleosil C18 7.0**	**IAM 2.5**	**IAM 7.0**	**Nucleosil OH 2.5**	**Nucleosil OH 7.0**	**TE**	**BE**	**AIE**	**EE**	**ECC**	**HF**	**EHOMO**	**ELUMO**	**EN**	**HARD**	**MAX_NEG**	**MAX_POS**	**DELTA**	**TDM**	**SA**	**V**	**HE**	**LOG_P**	**R**	**P**
**1**	2.51	4.32	−0.19	1.82	−0.41	0.99	−125943	−6134	−119809	−1014808	888865	−8.1	−9.2	−0.81	−5.0	−4.2	−0.30	0.34	0.64	2.61	792	1385	−1.4	6.57	144	56
**2**	1.75	2.31	−0.49	1.23	−0.44	0.77	−122106	−5447	−116660	−986416	864310	−173.6	−8.8	−0.38	−4.6	−4.2	−0.40	0.36	0.76	4.99	635	1123	−8.6	−0.01	109	42
**3**	1.37	2.25	−0.58	0.40	−0.44	0.67	−116762	−5719	−111043	−975452	858691	−73.8	−8.6	−0.40	−4.5	−4.1	−0.37	0.30	0.67	1.15	660	1155	−4.7	2.4	114	45
**4**	0.60	1.59	0.17	0.61	−0.44	0.65	−92905	−5482	−87423	−793186	700281	16.3	−8.9	−0.10	−4.5	−4.4	−0.43	0.31	0.74	2.31	586	1041	−3.6	3.99	102	41
**5**	2.14	2.22	0.07	0.03	0.22	0.26	−109704	−4536	−105168	−816705	707002	−109.6	−9.0	−0.61	−4.8	−4.2	−0.35	0.36	0.71	9.67	545	939	−6.7	−3.48	91	34
**6**	2.62	2.69	0.14	0.09	0.22	0.31	−131466	−5762	−125704	−1089747	958282	−175.4	−9.1	−0.88	−5.0	−4.1	−0.39	0.57	0.95	7.11	652	1154	−7.7	−2.89	112	42
**7**	2.77	2.86	0.23	0.17	0.23	0.32	−120482	−5379	−115103	−991599	871116	−127.8	−9.0	−0.69	−4.9	−4.2	−0.38	0.57	0.95	8.38	600	1062	−3.9	−2.27	105	40
**8**	2.40	2.51	0.17	0.12	0.23	0.31	−113306	−4827	−108480	−856446	743139	−125.3	−9.1	−0.82	−5.0	−4.1	−0.38	0.57	0.95	7.18	570	981	−7.0	−3.14	96	36
**9**	0.56	0.57	−0.13	−0.15	0.22	0.24	−85329	−3839	−81490	−550132	464803	−49.2	−9.0	−0.36	−4.7	−4.3	−0.36	0.41	0.77	4.44	515	837	−1.2	−0.14	74	28
**10**	1.68	2.17	−0.53	1.12	−0.43	0.74	−97523	−5382	−92141	−768876	671353	−95.0	−9.3	−0.36	−4.8	−4.5	−0.31	0.28	0.59	4.72	589	1050	−5.4	3.4	100	39
**11**	1.79	2.15	−0.58	0.61	−0.43	0.68	−130850	−7144	−123706	−1123270	992420	−85.9	−8.5	0.12	−4.2	−4.2	−0.26	0.15	0.41	4.69	798	1394	−7.6	5.05	133	58

**Table 3.** Summary of the results of principal component analysis derived for the compounds studied.

**No. of factor**	**Structural parameters^a^**			**HPLC retention data^b^**			**All data^c^**		

	**Eigen-value**	**Variance explained (%)**	**Total variance explained (%)**	**Eigen-value**	**Variance explained (%)**	**Total variance explained (%)**	**Eigen-value**	**Variance explained (%)**	**Total variance explained (%)**
**1**	9.24	46.18	46.18	3.42	56.92	56.92	11.34	43.60	43.60
**2**	5.82	29.10	75.27	1.98	33.08	90.00	7.59	29.21	72.81
**3**	2.41	12.07	87.34	0.46	7.61	97.61	3.20	12.30	85.11
**4**	1.16	5.81	93.15	0.10	1.67	99.28	1.34	5.16	90.27
**5**	0.74	3.68	96.83	-	-	-	1.16	4.46	94.73
**6**	-	-	-	-	-	-	0.74	2.86	97.59

a PCA performed only for structural parameters;

b PCA performed only for HPLC retention data;

c PCA performed for structural parameters along with HPLC retention data.

**Table 4.** Principal component analysis loadings by structural parameters.

**Structural parameters**	**Factor 1**	**Factor 2**
TE	**0.7651**	−0.6392
BE	**0.9569**	0.0165
AIE	**0.7404**	−0.6655
EE	**0.8450**	−0.4976
ECC	−**0.8492**	0.4809
HF	−0.0313	−**0.7792**
EHOMO	−0.4810	−0.2329
ELUMO	−0.2627	−**0.7643**
EN	−0.3883	−0.5919
HARD	−0.3389	**0.7326**
MAX_NEG	−0.5966	−0.2413
MAX_POS	0.4951	**0.7760**
DELTA	0.5749	**0.7005**
TDM	0.3436	0.6671
SA	−**0.9666**	−0.0155
V	−**0.9749**	0.0133
HE	0.2474	−0.4908
LOG_P	−0.5978	−**0.7178**
R	−**0.9384**	0.0650
P	−**0.9750**	−0.0713

**Table 5.** Principal component analysis loadings by HPLC retention data.

**HPLC retention data**	**Factor 1**	**Factor 2**
Nucleosil C18 2.5	−0.0666	−**0.9490**
Nucleosil C18 7.0	0.4324	−**0.8885**
IAM 2.5	−**0.7218**	−0.3973
IAM 7.0	**0.9270**	−0.2296
Nucleosil OH 2.5	−**0.9296**	−0.2897
Nucleosil OH 7.0	**0.9896**	−0.0226

**Table 6.** Principal component analysis loadings by all data.

**All data**	**Factor 1**	**Factor 2**
Nucleosil C18 2.5	−0.2015	−**0.9114**
Nucleosil C18 7.0	−0.5561	−0.6065
IAM 2.5	0.6107	−0.4332
IAM 7.0	−**0.7057**	0.1657
Nucleosil OH 2.5	**0.7475**	−0.5362
Nucleosil OH 7.0	−**0.8065**	0.3277
TE	0.6488	**0.7362**
BE	**0.9308**	0.1287
AIE	0.6210	**0.7579**
EE	**0.7461**	0.6032
ECC	−**0.7525**	−0.5869
HF	−0.1472	0.6855
EHOMO	−0.4196	0.2353
ELUMO	−0.3112	**0.7591**
EN	−0.3914	0.5898
HARD	−0.1857	−**0.7573**
MAX_NEG	−0.6159	0.0915
MAX_POS	0.5959	−**0.7068**
DELTA	0.6618	−0.5966
TDM	0.4756	−0.6349
SA	−**0.9554**	−0.1519
V	−**0.9681**	−0.1769
HE	0.1498	0.4308
LOG_P	−**0.7316**	0.5915
R	−**0.9413**	−0.2332
P	−**0.9753**	−0.0994

**Table 7.** Principal component analysis scores of the studied compounds.

**Compound**		**Structural parameters^a^**		**HPLC retention data^b^**		**All data^c^**	

**No.**	**Name**	**Factor 1**	**Factor 2**	**Factor 1**	**Factor 2**	**Factor 1**	**Factor 2**
**1**	amiodarone	−1.1793	−0.1644	1.5754	−1.5335	−1.4266	−0.2749
**2**	amlodipine	−0.2667	0.4491	1.0088	0.2542	−0.4023	−0.1355
**3**	diltiazem	−0.5703	−0.3440	0.5879	0.7398	−0.5999	0.4120
**4**	disopyramide	0.4344	−1.3892	0.1143	1.0613	0.2651	1.4139
**5**	nifedipine	0.7363	0.3826	−0.9964	−0.3150	0.8611	−0.3602
**6**	nimodipine	−0.1657	1.7114	−0.9049	−0.9047	0.1784	−1.6478
**7**	nisoldipine	0.2700	1.0449	−0.8930	−1.1573	0.4737	−1.1303
**8**	nitrendipine	0.7518	1.0173	−0.9329	−0.7018	0.8768	−0.9184
**9**	pentoxyphylline	1.6061	−1.0869	−1.1599	1.6600	1.5499	1.3053
**10**	propaphenone	0.4022	−0.9245	0.9337	0.4110	0.0136	0.9640
**11**	verapamil	−2.0187	−0.6963	0.6671	0.4860	−1.7899	0.3719

a PCA performed only for structural parameters;

b PCA performed only for HPLC retention data;

c PCA performed for structural parameters along with HPLC retention data.
